# Zika emergence, persistence, and transmission rate in Colombia: a nationwide application of a space-time Markov switching model

**DOI:** 10.1038/s41598-024-59976-7

**Published:** 2024-05-01

**Authors:** Laís Picinini Freitas, Dirk Douwes-Schultz, Alexandra M. Schmidt, Brayan Ávila Monsalve, Jorge Emilio Salazar Flórez, César García-Balaguera, Berta N. Restrepo, Gloria I. Jaramillo-Ramirez, Mabel Carabali, Kate Zinszer

**Affiliations:** 1 Université de Montréal, École de Santé Publique, Montreal, H3N 1X9 Canada; 2 Centre de Recherche en Santé Publique, Montreal, H3N 1X9 Canada; 3 Department of Epidemiology, Biostatistics and Occupational Health, McGill University, Montreal, H3A 1G1 Canada; 4 Universidad Cooperativa de Colombia, Faculty of Medicine, Villavicencio, 500003 Colombia; 5 Instituto Colombiano de Medicina Tropical, Universidad CES, Medellín, 055450 Colombia; 6 Infectious and Chronic Diseases Study Group (GEINCRO), San Martín University Foundation, Medellín, 050031 Colombia

**Keywords:** Epidemiology, Statistics, Infectious diseases

## Abstract

Zika, a viral disease transmitted to humans by *Aedes* mosquitoes, emerged in the Americas in 2015, causing large-scale epidemics. Colombia alone reported over 72,000 Zika cases between 2015 and 2016. Using national surveillance data from 1121 municipalities over 70 weeks, we identified sociodemographic and environmental factors associated with Zika’s emergence, re-emergence, persistence, and transmission intensity in Colombia. We fitted a zero-state Markov-switching model under the Bayesian framework, assuming Zika switched between periods of presence and absence according to spatially and temporally varying probabilities of emergence/re-emergence (from absence to presence) and persistence (from presence to presence). These probabilities were assumed to follow a series of mixed multiple logistic regressions. When Zika was present, assuming that the cases follow a negative binomial distribution, we estimated the transmission intensity rate. Our results indicate that Zika emerged/re-emerged sooner and that transmission was intensified in municipalities that were more densely populated, at lower altitudes and/or with less vegetation cover. Warmer temperatures and less weekly-accumulated rain were also associated with Zika emergence. Zika cases persisted for longer in more densely populated areas with more cases reported in the previous week. Overall, population density, elevation, and temperature were identified as the main contributors to the first Zika epidemic in Colombia. We also estimated the probability of Zika presence by municipality and week, and the results suggest that the disease circulated undetected by the surveillance system on many occasions. Our results offer insights into priority areas for public health interventions against emerging and re-emerging *Aedes*-borne diseases.

## Introduction

**Figure 1:**
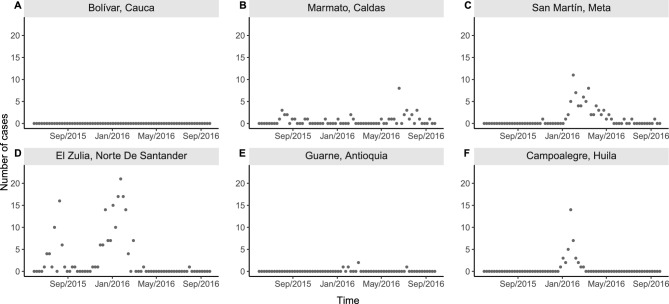
Examples of different temporal patterns of Zika reported cases counts by epidemiological week of first symptoms in selected municipalities of Colombia, epidemiological weeks 22/2015 to 39/2016: (**A**) disease observed always absent in Bolívar (above 1800m of altitude and average temperature of 20 ^∘^C); (**B**) few cases reported intermittently in Marmato (above 1300m, 22 ^∘^C); (**C**) an observed emergence with very few cases followed by observed extinction and observed re-emergence with more cases being reported in San Martín (below 500m, 27 ^∘^C); (**D**) an early emergence followed by observed extinction and subsequent re-emergence in El Zulia (below 200m, 27 ^∘^C); (**E**) few and sporadic cases being reported in Guarne (above 2000m, 18 ^∘^C); and (**F**) an observed emergence followed by observed extinction in Campoalegre (around 500m, 24 ^∘^C). Data source: Colombian National Public Health Surveillance System—*Sistema Nacional de Vigilancia en Salud Pública* (SIVIGILA).

Zika virus emerged in several tropical countries and territories, causing large epidemics between 2014 and 2016^1^. Identical to dengue and chikungunya viruses, Zika is transmitted to humans by the bite of infected *Aedes* mosquitoes, mainly *Aedes aegypti*^1,2^. These diurnal mosquitoes are well adapted to urban settings, live in intradomicile and peridomicile spaces, reproduce in small collections of fresh water, and are climate-sensitive^3–5^. At the population-level, characteristics that vary in space (e.g. elevation) and in space and time (e.g. temperature) impact *Aedes aegypti*’s presence, density, activity, and competence to transmit viruses^5–9^. Hence, these characteristics likely play a role in the spatio-temporal distribution of *Aedes*-transmitted diseases such as Zika.

Among the various space- and time-varying characteristics at the population level, warmer temperatures have been associated with an increased risk of *Aedes*-borne diseases^10–14^. Up to 35 ^∘^C, the increase in temperature increases the speed of *Aedes aegypti*’s life cycle (resulting in mosquito population growth) and the biting rates, while also decreasing the extrinsic incubation period (which is the time taken between the mosquito ingesting the virus and becoming infectious)^5,8^. By contrast, Zika transmission and vector competence are drastically reduced below 20 ^∘^C^8,9^. Humidity has been associated with the *Aedes aegypti* activity, survival and reproductive activity^15,16^. Rainfall can fill small containers, such as any recipient from uncollected waste with fresh water, forming ideal breeding sites for the *Aedes* mosquitoes to lay their eggs and live during the immature stages^15^. For that reason, improper waste management also plays an important role in the risk of diseases transmitted by *Aedes aegypti*, together with other factors that can increase the risk of exposure (e.g., housing materials, household crowding, access to piped water), particularly in areas with lower socioeconomic conditions^10,17–19^. Finally, higher human population densities increase the chances of mosquito-virus-human interaction^20,21^.

One of the countries most-affected by *Aedes*-borne diseases in South America is Colombia. Colombia has been endemic for dengue for decades, and the first Zika cases in the country were reported in August 2015^22,23^. Colombia has a very diverse geography, including islands, deserts, forests and mountain regions. Sociodemographic conditions also vary greatly within the territory. A robust National Surveillance System (*Sistema Nacional de Vigilancia en Salud Püblica* - SIVIGILA) is implemented in the country, where all cases seeking care in health facilities with a suspected or confirmed diagnosis of Zika are to be reported^24^. SIVIGILA data have been used to study the spatio-temporal patterns of dengue, Zika, and chikungunya in Colombia. However, most studies focused on a given municipality, department or both^17,25–33^, and only a few considered the data for the whole country^23,34–38^. Among these, the studies applying statistical models^34–36^ used data at the department level.

One reason for the lack of nationwide spatio-temporal studies at the municipal level in Colombia is that across the territory, the distribution of *Aedes*-borne diseases is highly heterogeneous. Figure 1 displays examples of the different temporal patterns of Zika-reported cases that can be observed across various geographically distinct Colombian municipalities. In some municipalities, there have never been reports of Zika cases. In others, the reported disease cases alternate between long periods of zero cases followed by long periods of cases which are often interspersed with zero cases and occur at different magnitudes over time. This complex type of data structure does not fit with most conventional statistical count models, such as Poisson or negative binomial regression models, due to the large number of zeroes^39^. Also, while zero-inflated models can inflate the probability of observing a zero count^40^, they would likely not be able to explain well the excessive numbers of consecutive zeroes^41^.

To address issues, like consecutive zeroes, with applying existing zero-inflated approaches to spatio-tempoal infectious disease counts, (^42^) proposed a zero-state Markov switching model. They assumed the disease switches between periods (i.e. *states*) of presence and absence in each area through a series of Markov chains. This typically means that when the disease is in a given state, it is more likely to remain in that state. Therefore, their model can produce long strings of zeroes and long periods of positive counts, interspersed with zeroes, as is commonly observed in spatio-temporal counts of infectious disease cases. Also, unlike existing zero-inflated models, this approach distinguishes between the re-emergence (absence to presence) and persistence (presence to presence) of the disease which is epidemiologically justified in many instances^42,43^. Our approach, explained in more detail below, extends the one by (^42^) by adding an initial absence period to their model as we are modeling the initial introduction of Zika in Colombia.

**Figure 2:**
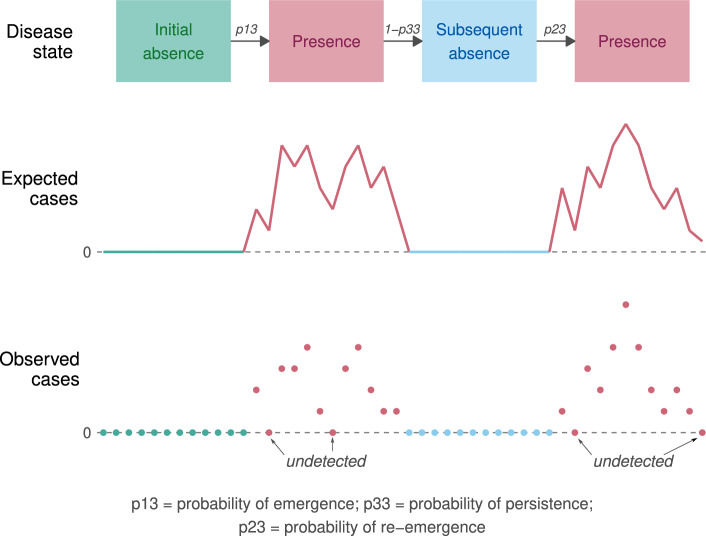
Diagram presenting the basic model structure considering three disease states: initial absence, presence, and subsequent absence.

A diagram presenting our basic model structure is given in Fig. 2. We assume that Zika switches between periods of initial absence, presence, and subsequent absence in each of the municipalities according to spatially and temporally varying probabilities of Zika emergence, re-emergence, and persistence. These probabilities, along with the transmission intensity rate when Zika is present, can depend on environmental and sociodemographic factors. Zero reported cases can arise from the absence states, representing the true absence of the disease, or from the presence state, representing undetected Zika. An important aspect of our approach is that we can calculate the probability that a zero arose from the *absence states* or the *presence state*^42^, and can therefore investigate where and when the disease was circulating undetected.

In this nationwide spatio-temporal ecological study, we analyzed the surveillance counts of Zika-reported cases by week between 31/May/2015 and 01/October/2016 in all 1,121 municipalities of Colombia obtained from SIVIGILA. With the proposed model described above, we aimed to unravel the Zika epidemic in Colombia by investigating its distribution and sociodemographic and environmental contributors by considering different aspects of the epidemic: the initial emergence of Zika in each municipality, the persistence of disease presence, the re-emergence, and the transmission intensity when the disease was present.

## Methods

In this ecological study, we analyzed the counts of Zika reported cases by municipality and week obtained from the Colombian National Public Health Surveillance System (*Sistema Nacional de Vigilancia en Salud Püblica* - SIVIGILA).

### Study site

**Figure 3:**
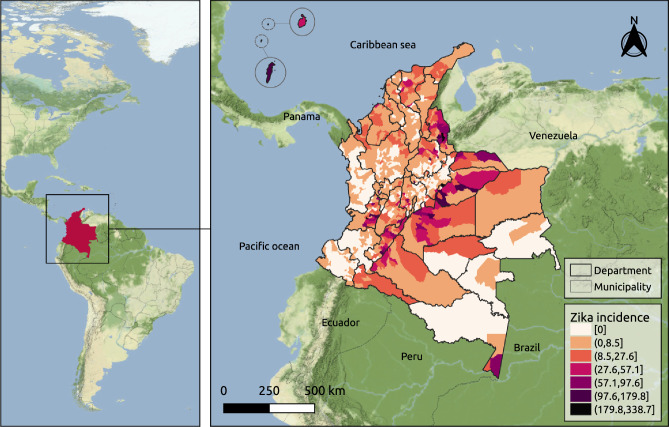
Localization of Colombia in the Americas and map of Colombia with the municipalities and geographical departments limits and the cumulative incidence of reported Zika cases per 10,000 inhabitants by municipality of residence from epidemiological weeks 22/2015 to 39/2016. Map created using QGIS (version 3.22) (QGIS.org, 2021. QGIS Geographic Information System. QGIS Association. http://www.qgis.org). Sources: Colombian National Administrative Department of Statistics—DANE—geoportal. Colombian National Public Health Surveillance System - SIVIGILA. Background map tiles by Stamen Design, under CC BY 3.0. Data by OpenStreetMap, under ODbL.

Colombia is located in South America and has 1,141,748 km^2^ and approximately 50.4 million inhabitants. The Colombian territory is divided into 1121 municipalities that are grouped into 33 departments (Fig. 3 and Supplementary Fig. S1). The country borders with other five countries (Panama, Venezuela, Brazil, Peru and Ecuador), in addition to the Pacific Ocean to the west and the Caribbean Sea of the Atlantic Ocean to the north. Colombia’s geography is very diverse and can be classified into six main natural regions: the Andes mountains, the Pacific coast, the Caribbean coast, the Llanos (savanna), the Amazon rain forest, and the insular area. Colombian climate is considered tropical and varies across its natural regions.

### Zika cases data

We obtained Zika-reported case data in all of Colombia from the SIVIGILA portal^44^. We aggregated the cases by the epidemiological week of symptoms onset and municipality of residence. The resulting cases dataset comprises the counts of probable and confirmed Zika cases over 70 epidemiological weeks and 1,121 Colombian municipalities during the period of the first Zika epidemic in the country (between 31/May/2015 and 01/October/2016). The cumulative incidence of reported Zika cases per 10,000 inhabitants for the study period is depicted in Fig. 3.

SIVIGILA publicly provides surveillance data that comes from each department’s Secretariat of Health. Probable and confirmed cases of Zika treated in public and private health facilities must be reported following the protocols of the National Institute of Health of Colombia^24^. Complete case definitions for Zika are included in the Supplementary Material Text 1.

### Environmental and sociodemographic data

The elevation (in meters) of each municipality (see Supplementary Fig. S2A) was calculated for the centroid of the urban area using the packages sf (version 1.0-15)^45^ and elevatr (version 0.99.0)^46^ in R (version 4.3.2)^47^. The shapefile with the urban areas was obtained at the geoportal of the Colombian National Administrative Department of Statistics - *Departamento Administrativo Nacional de Estadística* (DANE)^48^. When a municipality had more than one urban area, we selected the *cabecera municipal*, which corresponds to the main urban area of the municipality and is where the majority of the most important services, such as the city hall, are located. Other environmental data used in this work, including the Normalized Difference Vegetation Index (NDVI), maximum temperature, accumulated precipitation and relative humidity, were previously organized by municipality and week and made publicly available by Siraj et al.^49,50^. The authors made available two datasets: one weighted by population density and one without population weighting and here, we chose without weighting given that population density was included as a covariate and we were interested in the environmental associations at the municipal level. We chose to use the second. For further information on the data processing methodology, please refer to Siraj et al.^50^.

From the data by epidemiological week and municipality, we calculated for each municipality the average NDVI for the entire study period (see Supplementary Fig. S2B). The NDVI quantifies vegetation greenness by using remote sensing technologies. In our study area, the NDVI ranged from 0.22 to 0.80. Areas with NDVI close to +1 have a higher possibility of having dense vegetation cover, while those with values close to 0 are more urban with little vegetation cover. For each epidemiological week and municipality, we obtained the maximum temperature in Celsius degrees (^∘^C, see Supplementary Figs. S2C and S3), the accumulated precipitation (in mm, see Supplementary Figs. S2D and S4) and the relative humidity (in %, see Supplementary Figs. S2E and S5). The population estimates for 2015 and 2016 were obtained at the DANE website^51^. We calculated the population density by dividing the population of each municipality by its area in km^2^ from a shapefile of the municipalities’ limits obtained at the DANE geoportal^48^. Supplementary Fig. S6A shows the map with the mean population density (2015-2016) by km^2^ for each municipality.

Finally, we obtained the percentage of the population with unsatisfied basic needs (UBN) from the national census of 2018^52^ (see Supplementary Fig. S6B). The UBN is an index provided by DANE that captures socioeconomic vulnerabilities such as inadequate housing conditions, overcrowded households, inadequate or no access to basic sanitation, children not attending school in the household, and households with elevated economic dependency^53^.

### Statistical model

Let $y_{it}$ denote the number of Zika cases reported in municipality $i=1, \dots , 1,121$ during week $t=1,\dots ,70$. We assumed that Zika could be in one of three disease states within each municipality *i* during each week *t* denoted by the indicator $S_{it}$,

$$\begin{aligned} S_{it}=\left\{ \begin{array}{ll} 1,&{} \text{ initial } \text{ absence }, \\ 2,&{} \text{ subsequent } \text{ absence }, \\ 3, &{} \text{ presence }. \end{array} \right. \end{aligned}$$

If at least one Zika case was reported, i.e. $y_{it}>0$, we assumed the disease was present, i.e. $S_{it}=3$. However, Zika may have been circulating undetected and as such, $S_{it}$ was treated as an unknown parameter in the model and estimated whenever $y_{it}=0$.

If Zika was present ($S_{it}=3$), we assumed the reported cases were generated by a negative binomial distribution, and if Zika was absent ($S_{it}=1$ or 2), no cases were reported,

1 $$\begin{aligned}&y_{it} \mid S_{it},y_{i(t-1)} \sim {\left\{ \begin{array}{ll} 0, &{} \text {if} S_{it} =1\text { or }2 \text{ (absence)} \\ { NB(\lambda _{it},r)}, &{} \text {if} S_{it} =3 \text { (presence)}, \end{array}\right. } \end{aligned}$$

where $\lambda _{it}$ is the expected number of reported cases given Zika was present and *r* is an overdispersion parameter so that the variance is given by $\lambda _{it}+\lambda _{it}^2/r$.

To capture the transmission process of Zika when it was present, we assumed that $\lambda _{it}$ follows an endemic/epidemic model^54^,

2 $$\begin{aligned} \lambda _{it}=\lambda _{it}^{AR}y_{i(t-1)}+\lambda _{i}^{BL}. \end{aligned}$$

In (2), $\lambda _{it}^{AR}$ represents the transmission intensity rate of Zika when it was present which was assumed to depend on a vector of spatio-temporal covariates $\varvec{x}_{it}$ through a mixed log-linear regression,

3 $$\begin{aligned} \log (\lambda _{it}^{AR})&= \beta _{0}^{AR}+b_{i}+c_{\text {dept}(i)}+\varvec{x}_{it}^T\varvec{\beta }, \end{aligned}$$

where $b_{i}$ and $c_{\text {dept}(i)}$ are zero-mean municipality and department-specific normal random intercepts; $\beta _{0}^{AR}$ is the overall intercept, representing the log of the transmission intensity rate in an average municipality and department when all covariates are 0; and $\varvec{\beta }$ is a vector of covariate coefficients. In (2), $\lambda _{i}^{BL}$ is the baseline component representing the expected number of reported cases when no cases were reported in the previous week. We assumed $\lambda _{i}^{BL}$ can vary between municipalities, so that, $\log (\lambda _{i}^{BL})=\beta _{0}^{BL}+d_{i}$, where $d_{i}$ is a zero-mean municipality specific random intercept and $\beta _{0}^{BL}$ is the overall intercept.

As we would generally expect periods of Zika presence and absence to last several consecutive weeks, we used a Markov chain for modeling the switching between the states. Therefore, the current disease state in municipality *i* $S_{it}$ only depends on the previous state $S_{i(t-1)}$ and potentially on the covariates and random effects. The probabilities of transitioning from $S_{i(t-1)}=j$ to $S_{it}=k$ for $j,k=1,2,3$ are given in the following within-area transition matrix,4The probability of Zika emerging in municipality *i* during week *t*, $p13_{it}$ in (4), was assumed to depend on a vector of space-time covariates $\varvec{z}_{it}$ through a mixed logistic regression,

5 $$\begin{aligned} \text {logit}(p13_{it})= \eta _{0}+e_{\text {dept}(i)}+\varvec{z}_{it}^T\varvec{\eta }, \end{aligned}$$

where $e_{\text {dept}(i)}$ is a zero-mean department specific normal random intercept; $\eta _{0}$ is the overall intercept, representing the logit of the probability of Zika emerging in an average department when all covariates are 0; and $\varvec{\eta }$ is a vector of covariate coefficients. As the disease states $S_{it}$ and $S_{i(t-1)}$ were not known when zero cases were reported, often the transition of (4) was not observable. Therefore, we did not include municipality-specific random intercepts in the transition probabilities as it made model fitting unstable due to the amount of missing information.

The probability of Zika re-emergence, $p23_{it}$ in (4), was assumed to be equal to the probability of emergence plus a shift in intercept,

6 $$\begin{aligned} \text {logit}(p23_{it})= \text {logit}(p13_{it})+(\gamma _0+f_{\text {dept}(i)}-\eta _0-e_{\text {dept}(i)}), \end{aligned}$$

where $f_{\text {dept}(i)}$ is a zero-mean department-specific normal random intercept and $\gamma _0-\eta _0$ represents the difference, on the logit scale, between the probability of Zika re-emergence and emergence in an average department when all covariates are 0. The shift in intercept was needed to account for the study start date. For example, if the study began two weeks earlier, the probability of emergence would reduce but the probability of re-emergence would not change. Also, as the disease established itself at some point in time, there may have been a shift in conditions not entirely accounted for by the covariates.

Finally, the probability of Zika persistence, $p33_{it}$ in (4), was modeled similarly to emergence but the covariates, $\varvec{w}_{it}$, and their effects were allowed to differ,

7 $$\begin{aligned} \text {logit}(p33_{it})= \zeta _{0}+g_{\text {dept}(i)}+\varvec{w}_{it}^T\varvec{\zeta }, \end{aligned}$$

where $g_{\text {dept}(i)}$ is a zero-mean department-specific normal random intercept, $\zeta _{0}$ is the overall intercept and $\varvec{\zeta }$ is a vector of covariate coefficients.

### Model specification

The relative humidity and the maximum temperature were included in the model lagged by one week to account for the time elapsed, on average, between an infected mosquito biting a person and the onset of symptoms^55^. The weekly accumulated precipitation was lagged by four weeks to account for the additional time needed for a possible increase in the *Aedes* mosquito population after a rainy week^56,57^.

All covariates were included in the emergence (5), re-emergence (6), and transmission intensity rate (3) equations. For the transmission intensity rate, we also considered the cumulative incidence of Zika up to four weeks prior and its square to account for the potentially non-linear effect of the depletion of the susceptible population on the transmission intensity rate. The four-week lag was defined considering the average time elapsed between the person getting bitten by an infected mosquito and developing immunity against the Zika virus^55,58,59^.

The largest factor affecting Zika persistence is likely the previous number of cases as the disease would continue to be present when there are many infectious individuals. Therefore, due to a high amount of multicollinearity, we only included the previous week’s cases and the population density as covariates potentially associated with Zika persistence.

The population density and the previous week’s cases were log-transformed to reduce high skewness. All covariates were standardized to improve the mixing of our Bayesian algorithm and to facilitate comparisons between covariate’s magnitude of association.

### Model fitting

To fit the model (1)–(7), we followed the Bayesian approach described in (^42^). When there were no cases reported by a municipality, the disease state of the model was not known as Zika could have been either absent, initially or subsequently, or present but undetected by the surveillance system. To help quantify uncertainty in the unknown disease states whenever zero cases were reported by a municipality, we calculated the posterior probability, i.e. the probability given all observed data, of Zika initial absence, presence, and subsequent absence^42^.

An initial distribution for the disease state in week 1 was required to fit the model. If a municipality reported a positive number of cases in week 1, then we knew Zika was present, otherwise, we assumed there was a 5% chance Zika was present but undetected and a 95% chance Zika was initially absent.

We fitted the models using NIMBLE (version 1.0.1)^60–62^ in R (version 4.3.2)^47^. Wide priors were assumed for all model parameters. Convergence was checked using the Gelman-Rubin statistic (all estimated parameters$<1.03$), the minimum effective sample size ($>2000$) and by visually examining the traceplots^63^. For the maps and graphs, we used ggplot2 (version 3.4.4)^64^ and colorspace (version 2.1-0)^65^ in R (version 4.3.2)^47^. Codes are available in https://doi.org/10.5281/zenodo.10651575.

To check model fit, we assessed plots of fitted values compared to observed values. Broadly, the fitted values were constructed through simulation from the fitted model. See the Supplementary Material Text 2 for more details about model fitting, estimation of the unknown disease states, and the fitted values.

### Ethical considerations

This study was approved by the Science and Health Research Ethics Committee (*Comité déthique de la recherche en sciences et en santé* - CERSES) of the University of Montreal, approval number CERSES-19-018-D.

**Figure 4:**
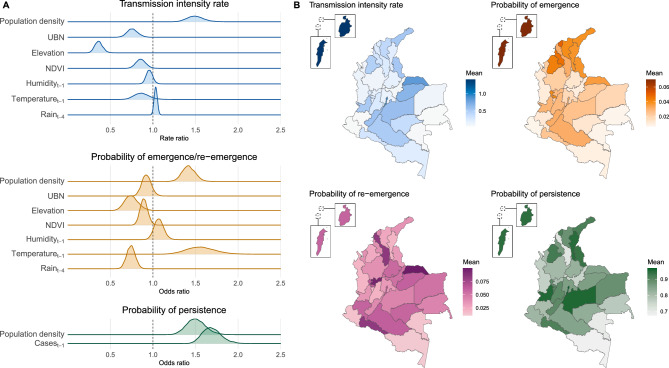
Association of covariates with the Zika epidemic in Colombia, epidemiological weeks 22/2015 to 39/2016. (**A**) Posterior distribution of the rate ratio/odds ratio associated with standardized covariates* and the transmission intensity rate, probabilities of emergence and re-emergence, and probability of persistence of Zika, and (**B**) posterior mean of the transmission intensity rate, probability of emergence, re-emergence, and persistence of Zika by department after adjusting for the department-specific random effect and the average values of the covariates in the department. Maps created using R (version 4.3.2, https://www.r-project.org/). *Population density and cases_t-1_ are log transformed. UBN = percentage of the population with unsatisfied basic needs. NDVI = Normalized Difference Vegetation Index.

## Results

Between epidemiological weeks 22/2015 and 39/2016, there were 72,031 Zika cases reported to the Colombian national surveillance system. The first cases were residents of three municipalities located in the department of Norte de Santander (San José de Cücuta, El Zulia and Puerto Santander), at EW 26/2015. Valle del Cauca, Norte de Santander, and Santander were the departments with the highest number of reported cases (20,965, 8,666 and 8,659, respectively) (see Supplementary Fig. S7). During the study period, 348 (31%) municipalities did not report Zika cases (Supplementary Table S1). The departments with a higher percentage of municipalities not reporting Zika cases were Guainía, Vaupés, Nariño, Chocó, Amazonas and Boyacá. Higher cumulative incidences per 10,000 inhabitants were observed in municipalities south of the Colombian Andes and the departments of Norte de Santander, Valle del Cauca and Archipelago of San Andrés, Providencia, and Santa Catalina (Fig. 3).

### Zika association with covariates

Figure 4A shows the posterior distribution of the transmission intensity rate ratio (TRR) and odds ratio (OR) associated with the covariates in standardized form.

Assuming a 95% credible interval (CI), when Zika was present, transmission was more intense in areas with higher population density (TRR mean 1.50, 95%CI 1.32;1.70), lower percentage of the population with unsatisfied basic needs (TRR 0.76, 95%CI 0.64;0.89), lower altitude (TRR 0.36, 95%CI 0.28;0.46), and/or less vegetation cover (TRR 0.86, 95%CI 0.75;0.98). It is worth mentioning that the transmission intensity rate showed a borderline direct association with the weekly accumulated rain (TRR 1.03, 95%CI 1.00;1.07, lagged by four weeks).

Supplementary Fig. S8 shows the association between cumulative incidence (lagged by four weeks) and the Zika transmission intensity rate by department after adjusting for the department-specific random effect and the average values of the covariates in the department. A rapid decrease in the transmission intensity rate was estimated with the increase of the cumulative incidence up to 5 cases per 10,000 inhabitants, more apparent for the department of Archipelago of San Andrés, Providencia and Santa Catalina. Above 5 cases per 10,000 inhabitants, the transmission intensity rate slightly decreases with the increase of the cumulative incidence.

Zika emerged and re-emerged sooner in municipalities with lower altitude (OR mean 0.74, 95%CI 0.60;0.92), less vegetation cover (OR 0.89, 95%CI 0.81;0.98) and/or less weekly accumulated rain (OR 0.74, 95%CI 0.64;0.84, lagged by four weeks). Zika also emerged and re-emerged sooner in more densely populated municipalities (OR 1.42, 95%CI 1.27;1.57) and/or with higher weekly maximum temperatures (OR 1.56, 95%CI 1.26;1.92, lagged by one week).

The disease persisted for longer in more densely populated municipalities (OR mean 1.50, 95%CI 1.30;1.74). Controlled by the population density, Zika was more likely to persist in a municipality when and where a higher number of cases was reported in the previous week (OR 1.69, 95%CI 1.50;1.94).

### Department-specific transmission intensity rate and probabilities of emergence, re-emergence, and persistence

After adjusting for the department-specific random effects and average values of the covariates in each department, higher transmission intensity rates were estimated in the Archipelago of San Andrés, Providencia and Santa Catalina, followed by departments located south of the Colombian Andes, Valle del Cauca, Norte de Santander, and in the north of the country (Fig. 4B and Supplementary Fig. S9). A similar spatial pattern was observed for disease emergence, but departments in the north of the country showed, on average, a higher probability of emergence compared to departments south of the Andes. However, it is worth mentioning that the differences in the probability of emergence across the departments were very small (see Supplementary Fig. S10). The same is true for the probability of re-emergence. Zika was more likely to persist in the departments of Archipelago of San Andrés, Providencia and Santa Catalina, Meta, Bogotá D.C, Valle del Cauca, Córdoba, Cesar, Norte de Santander, La Guajira, and Putumayo (Fig. 4B Supplementary Fig. S10).

### Zika transmission intensity rate

Maps showing the evolution of the transmission intensity rate over the entire study period are displayed in Supplementary Movie S1. The transmission intensity rate generally declined over the study period as the susceptible population becomes depleted. The contribution of the covariates can be seen in the spatial variation of the maps as municipalities located in the mountains typically experience much lower transmission of Zika. Additionally, heterogeneity introduced by the random effects is also clearly visible. For example, there are certain municipalities on the Pacific coast (in the departments of Chocó, Cauca, and Nariño) with very low transmission rates of Zika despite being at a low elevation. At the beginning of the study period, the highest Zika transmission rates were observed in the municipalities of San Andrés (Archipelago of San Andrés, Providencia and Santa Catalina department), Guadalajara de Buga (Valle del Cauca), Tauramena (Casanare), Bucaramanga (Santander), and Saravena (Arauca).

Supplementary Fig. S11 depicts the posterior mean of the baseline expected number of reported cases by municipality, representing the expected number of reported cases in a given week if no cases were reported in the previous week. It ranged from 0.13 in González (Cesar department) to 2.86 in Chaguaní (Cundinamarca department). Higher baselines were mainly observed in some municipalities from the departments of Cundinamarca, Magdalena, Norte de Santander, Valle del Cauca, Córdoba, Tolima, Antioquia, Casanare, and Santander.

### Fitted values and posterior probability of Zika presence

**Figure 5:**
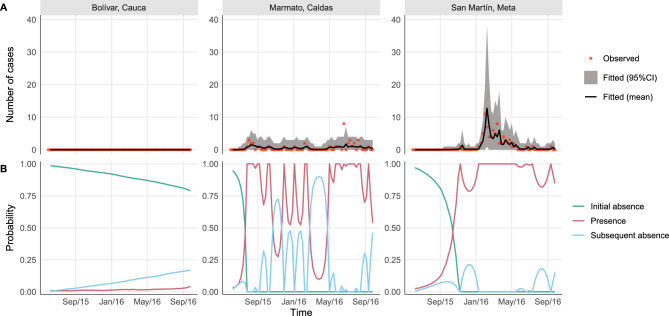
Fitting of the number of Zika cases and posterior probability of each disease state by epidemiological week (EW) in three selected municipalities, EWs 22/2015 to 39/2016, Colombia. (**A**) Observed versus fitted number of Zika cases (mean and 95% Credible Interval—CI) and (**B**) posterior probability of initial absence, presence, and subsequent absence of Zika.

For Fig. 5, we selected the first three municipalities from Fig. 1 as examples to compare the observed number to the fitted number of Zika cases (first row) and the estimated posterior probabilities of each disease state (second row).

As can be seen in the first row of Fig. 5, the fitted model was able to reproduce the reported number of cases well. The fitted mean follows the observed trend and the 95% credible intervals capture the majority of the observations. Note for the second row of Fig. 5, that when cases were reported by a municipality, the posterior probability of Zika being present was always 1. Otherwise, if zero cases were reported, the disease could have either been absent, initially (probability given by green line) or subsequently (blue line), or present but undetected (red line). In Marmato and San Martín, the posterior probability of Zika presence began to increase noticeably several weeks before the first reported cases and the posterior probability remained high when no cases were reported afterwards. In general, as the number of consecutive weeks with no cases being reported increased, the model becomes more certain that Zika is truly absent and not undetected. In Bolívar, which never reported any cases, the model estimated a 15% chance that Zika was no longer in the initial absence state in the last week, implying it may have circulated undetected at some earlier point in time. These patterns in the posterior probabilities were typical across the other municipalities.

**Figure 6:**
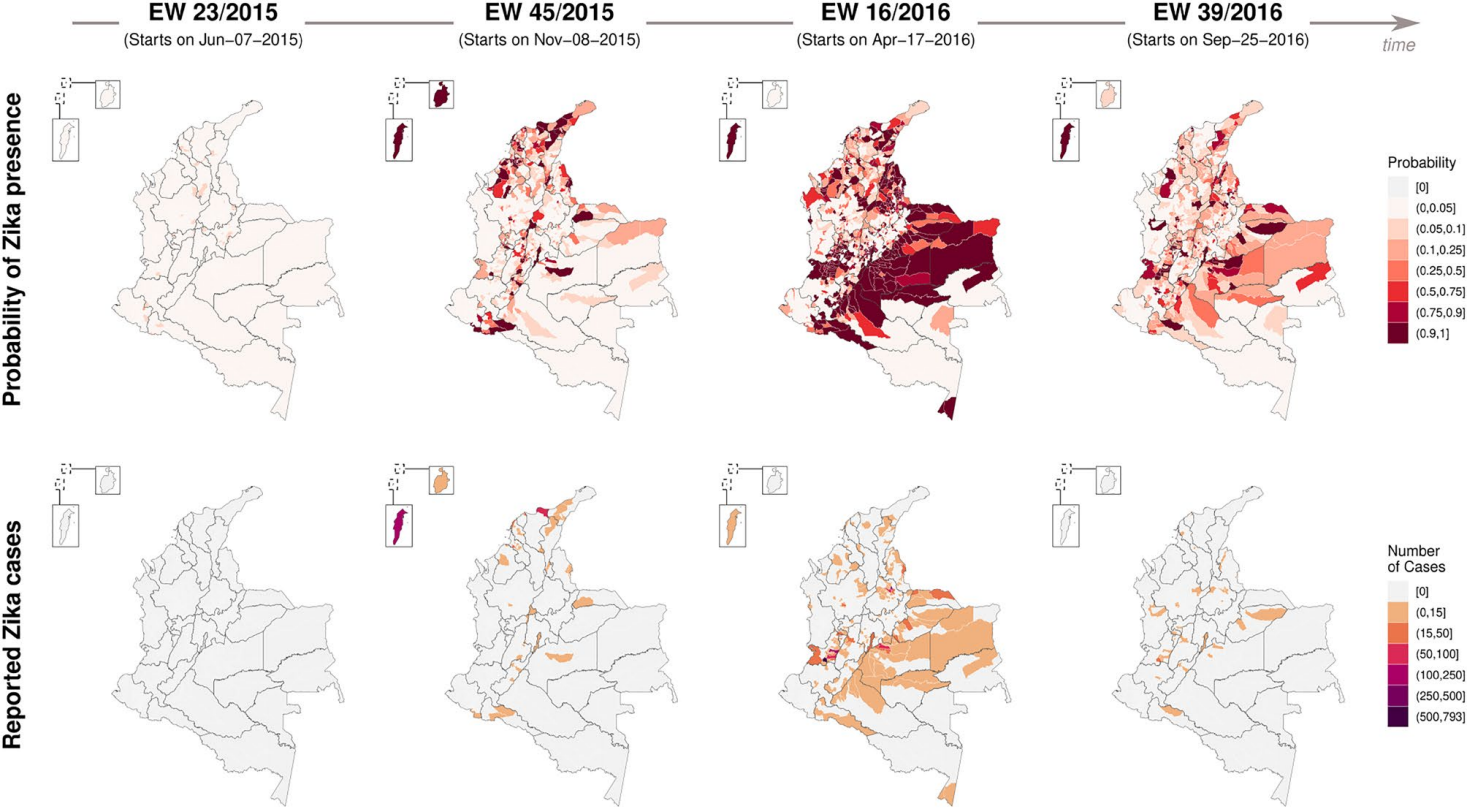
Posterior probability of Zika presence (first row) and observed number of reported Zika cases (second row) by municipality in four moments of time between epidemiological weeks (EWs) 22/2015 and 39/2016, Colombia. Maps created using R (version 4.3.2, https://www.r-project.org/).

In Fig. 6, we compared the maps with the posterior probability of Zika presence (first row) with the maps with the observed number of reported cases (second row) at four different moments during the study period. The maps for the entire study period are displayed in Supplementary Movie S2. When there are reported cases, the probability of Zika presence is always 1. However, we observed that the estimated probability of Zika presence increased before cases began to be reported, and remained high in certain municipalities, some of which had no reported cases of Zika. This was partly due to the department-specific random intercepts in the model, as municipalities with no cases would have an increased probability of Zika presence if other municipalities in the same department reported cases.

## Discussion

In this work, we studied the first Zika epidemic in Colombia using a novel modeling approach, which could manage the excessive amount of zeros in the data as well as the different temporal patterns of reported cases between the municipalities. The proposed model estimated the association of environmental and sociodemographic covariates with the probabilities of emergence, re-emergence, and persistence, and with the transmission intensity rate of Zika. Our results suggest that the population density, elevation, and maximum temperature were the main contributors to the first Zika epidemic in Colombia. We were also able to estimate the probability of Zika presence by week and municipality, which often increased before the first official report of Zika.

Population density had a strong positive association with the transmission intensity rate, the probabilities of emergence, re-emergence, and persistence of Zika (Fig. 4A). Although an emerging virus may enter a territory through a smaller city, it will likely establish itself more readily in larger cities with higher population densities, from where it may spread to the rest of the territory^23^. Densely populated areas provide more opportunities for the virus to infect vectors and humans.

We found an inverse association between the percentage of the population with unsatisfied basic needs and the Zika transmission intensity rate, and the direction of this association needs to be interpreted with caution. The majority of municipalities with higher percentages of their populations with unsatisfied basic needs belong to the following departments: Chocó, Nariño, Vichada, Guainía, Vaupés, Amazonas, and La Guajira (see Supplementary Fig. S6B). Among the 138 municipalities of these departments, 95 (or 68.8%) did not report any cases of Zika during the study period (Fig. 3). These particular locations are known to have problems of underreporting and the local populations face important barriers to access the health system. These can be explained by the presence of extensive jungle-covered areas, an ongoing violent conflict, a limited number of healthcare facilities, and potentially weaker surveillance systems compared to other areas. Nonetheless, the inverse association found could be a consequence of the spatial unit size used in the analysis. In Colombia, there are important social inequalities that cannot be fully captured by a single socioeconomic index value for an entire municipality. To better understand the role of socioeconomic conditions in the Zika epidemic, it may be necessary to work with smaller spatial units to have sufficient resolution to capture these inequalities.

Zika’s transmission intensity rate, probability of emergence and probability of re-emergence were higher in more urban municipalities, i.e., with less vegetation cover (represented by the NDVI), and/or with lower altitude (Fig. 4A). The inverse association with NDVI may be explained by the preference of *Aedes aegypti* for urban settings^66^, and also by the presence of heat islands in urban settings affecting the microclimate. The estimated effect of elevation and temperature on the transmission intensity rate needs to be interpreted with caution. It should not be concluded that there is no association between temperature and transmission intensity, only that, after accounting for elevation, the temperature effect was towards the null. As can be seen in Fig. S2 panels A and C, temperature and elevation had a strong inverse correlation, with areas with lower temperatures mostly found at higher elevations. Therefore, areas with low temperatures did experience lower transmission of Zika on average. Also, the temperature in Colombia remained relatively constant over time (see Supplementary Fig. S3), mainly varying spatially. Therefore, it is possible that elevation was capturing part of the association between the Zika transmission intensity rate and temperature.

Climate factors (temperature and rain) were strongly associated with the emergence and re-emergence of Zika but less so with its transmission intensity rate (Fig. 4A). In addition to the possible explanations discussed above, it is possible that there were interactions between different climate factors (including but not limited to temperature, humidity and rain) that would require more complex modeling structures to be captured. We hypothesize that climate factors, along with elevation and NDVI, captured the presence of the *Aedes aegypti* mosquitoes, which is necessary for local transmission to occur. Using climate data as proxies of the mosquitoes’ presence is advantageous, as such data tend to be more readily available than mosquito data.

An inverse association between rain and the probabilities of Zika emergence and re-emergence was found. During the study period (2015-2016), the El Niño event was affecting South America, causing warmer temperatures and droughts in Colombia and boosting the transmission of *Aedes*-borne diseases^67,68^. A negative association between precipitation and the number of dengue cases was found in a previous study evaluating the effects of local climate and El Niño in Colombia^68^. Analyzing data by department, Chien et al.^34^ found both positive and negative associations between rain and the risk of Zika in Colombia. Although rain may increase mosquito density by creating potential breeding sites, heavy rain can wash away the eggs and larvae. Also, droughts may result in people storing water in improvised containers inside their households as a response to water supply interruptions. These improvised water-filled containers are well-suited for *Aedes aegypti* breeding and generally favor an increase in mosquito density. An association between periods of drought and increased risk of *Aedes*-borne diseases has been previously described^14,69^.

The increase in the probability of disease presence weeks before the first reported cases indicates that Zika circulated undetected in the early phases of the epidemic. This is expected, considering that Zika was a new emerging disease and there was no active surveillance implemented to detect the entry of the virus into the country. The model presented in this study has the potential to be adapted for similar scenarios and provide insight to inform our understanding of emerging and re-emerging diseases’ spatio-temporal distributions and, ultimately, help guide control and prevention strategies. The results may also indicate locations with notifiable disease reporting issues when a high probability of disease presence is estimated but when no cases are reported.

We recommend the implementation of effective measures, including enhanced surveillance and vector-control strategies, in Colombian urban centers, particularly those with high population density. These measures are essential for mitigating the introduction of other emerging viruses transmitted by *Aedes*, as well as for reducing the burden of the *Aedes*-borne diseases that are endemic in the country. On the other hand, the processes of urbanization, resulting in increased population density and loss of vegetation cover, increase the risk of *Aedes*-transmitted diseases. Therefore, strategies for sustainable human development that prioritize the preservation and augmentation of vegetation cover should be considered to effectively prevent and control these diseases. Additionally, ensuring the population access to piped water without supply interruptions is crucial. When that is not feasible, education campaigns should focus on how to prevent breeding sites and appropriate containers provided to the community.

There are different limitations to this study. Zika cases are based on SIVIGILA notifications which is largely based on passive surveillance^24^. Hence, our study population included mostly patients who developed symptoms and sought health care. Also, the observed counts are likely underestimated due to underreporting, a common limitation when working with surveillance data, and that has been detected in Colombia’s surveillance system^70^. Dengue and chikungunya were circulating simultaneously prior to the onset of the Zika epidemic in Colombia^23^. During the study period, 142,008 dengue cases and 22,917 chikungunya cases were registered in SIVIGILA. The differential diagnosis between the three diseases can be challenging without laboratory confirmation, although there were protocols for differential clinical diagnosis between the three infections^71–73^. Among our study population, only 7.7% of Zika cases were laboratory-confirmed. In the same period, laboratory confirmation was obtained for 40.6% of dengue cases and 3.5% of chikungunya cases. There is the likelihood that there is misclassification error between the different diseases for clinically-based diagnosis. We aggregated the data based on the municipality of residence, although cases may have been infected elsewhere. The climate factors were included in the model with predefined lags, although we acknowledge that more complex structures considering different lags, interactions, and possibly nonlinear effects may be necessary to better capture the association between climate and *Aedes*-borne diseases^74^. Including such structures in the scope of the present work is challenging, considering the large number of model parameters, areas, and time points.

The range of the *Aedes aegypti* mosquito has been expanding as a consequence of global warming^75^. This is resulting in increased occurrence of *Aedes*-transmitted diseases in endemic areas but also the emergence in previously unaffected areas^76^. Large epidemics of Zika can occur in areas where the majority of the population is naive for previous infection. Although Zika is usually mild, infection during pregnancy can cause congenital malformation in the fetus and its risk should not be neglected^77^. Our model can be used to better understand the factors contributing to disease emergence, re-emergence, persistence, and transmission intensity at high spatial and temporal resolution and can be applied to different infectious diseases. This can provide valuable insights into the characteristics of areas that should be prioritized for interventions such as vector-control measures, enhanced surveillance, and public health campaigns.

### Supplementary Information

Supplementary Information 1.

Supplementary Information 2.

Supplementary Information 3.

## Data Availability

The data used in this study are secondary data and are publicly available. The data on Zika cases can be downloaded at the SIVIGILA website (http://portalsivigila.ins.gov.co/). Population data and the percentage of people with unsatisfied basic needs can be found at the DANE website (https://www.dane.gov.co/). Environmental data was organized and made available by Siraj et al.^49^ (https://doi.org/10.5061/dryad.83nj1). Processed data and codes are available in https://doi.org/10.5281/zenodo.10651575.
