# Supplementary material for: Zika emergence, persistence, and transmission rate in Colombia: a nationwide application of a space-time Markov switching model
Source: Sci Rep. 2024 May 1;14:10003. doi: 10.1038/s41598-024-59976-7 (PMC11063144; doi:10.1038/s41598-024-59976-7)
Supplement: Supplementary file 3 — Supplementary Information 3. [file 41598_2024_59976_MOESM3_ESM.pdf]

Supplementary Material for

Zika emergence, persistence, and transmission rate in Colombia: a nationwide application of a space-time Markov switching model

Laís Picinini Freitas*, Dirk Douwes-Schultz*, Alexandra M. Schmidt, Brayan S. Ávila Monsalve, Jorge Emilio Salazar Flórez, César García-Balaguera, Berta N. Restrepo, Gloria I. Jaramillo-Ramirez, Mabel Carabali, Kate Zinszer

***Corresponding Authors: Laís Picinini Freitas and Dirk Douwes-Schultz**
E-mail: lais.picinini.freitas@umontreal.ca, dirk.douwes-schultz@mail.mcgill.ca
***These authors contributed equally to this work.**

This PDF file includes:

Supplementary text
Figures S1 to S11
Table S1
Legends for Movies S1 to S2
SM References

Other supplementary materials for this manuscript include the following:

Movies S1 to S2

Supplementary Text

1. Zika case definition

The following criteria describes the case definition for Zika cases adopted by the Colombian National Institute of Health (NIH) (1).

Suspected case. Patient living in municipalities without confirmed Zika transmission presenting with a rash and one or more of the following symptoms not explained by other medical conditions: fever not greater than 38.5°C, nonpurulent conjunctivitis or conjunctival hyperemia, arthralgia, myalgia, headache, or malaise. Additionally, one of the following conditions: i) Person who visited, two weeks before the onset of symptoms, in countries or municipalities located between 0 and 2,200 m above sea level, with or without confirmed indigenous circulation of the Zika virus; ii) Person who had sexual contact without barrier protection two weeks before the onset of symptoms with a person who in the eight weeks prior to sexual contact visited areas with confirmed Zika transmission and/or areas with the presence of *Aedes* mosquitoes.

Case confirmed by clinical epidemiological criteria. Patient who had been in countries or municipalities located between 0 and 2,200 meters above sea level with confirmed autochthonous circulation of the Zika virus two weeks before the onset of symptoms and who presented a rash and one or more of the following symptoms that were not explained by other medical conditions: fever not higher than 38.5 °C, non-purulent conjunctivitis or conjunctival hyperemia, arthralgia, myalgia, headache or general malaise.

Case confirmed by laboratory. Case that met the definition for probable or confirmed by clinical epidemiological case and that presented a positive result for Zika virus by RT-PCR (or immunohistochemistry in histopathological analysis) performed at the National Reference Laboratory of the NIH or collaborating centers designated by the NIH.

Discarded case. A suspected case in which a laboratory test was carried out within the time established for the detection of the viral agent, and showed negative results for Zika virus, or another etiological diagnosis was established.

In addition to the case definition above, in our study we also adopted the following definition:

Probable case. A case meeting the definition of a suspected case that was not discarded.

2. Additional information regarding model fitting, estimation of the unknown disease states and the fitted values

We fitted the model using a hybrid Gibbs sampler, described in more detail in (2), which we implemented in the R package `NIMBLE` (3). Gibbs sampling begins by partitioning the unknown parameter vector into blocks and setting suitable random initial values for each block. Then the algorithm alternates between sampling each block of unknown parameters conditional on all other blocks and the observed data (4). After an initial burn-in period the Gibbs sampler draws correlated samples from the joint posterior distribution, i.e., distribution given the observed data, of all unknown parameters. Although, it is possible for the samples to be too correlated to draw reasonable inference from and the Gibbs sampler can get stuck in local modes, therefore, it is important to assess convergence of the algorithm (5). Note that the unknown state indicators, that is S_{it} for all i and t such that $y_{it} = 0$, are a part of the unknown parameter vector in our application as the disease state is not known when no cases are reported. We sampled each unknown parameter, which was not an unknown state indicator and did not have a conjugate prior, individually, using an adaptive random walk Metropolis step (6). We sampled all the unknown state indicators in a municipality jointly using a forward filtering backwards sampling (FFBS) algorithm (7). Sampling the unknown state indicators of a Markov switch model jointly is typically much more efficient than sampling them individually (8).

Once a sample from the joint posterior has been obtained, the posterior probability that the disease was in disease state s, for $s = 1$ (initial absence), $s = 2$ (subsequent absence) and $s = 3$ (presence), at time t in area i, assuming $y_{it} = 0$ so no cases were reported, is given by,

$$P(S_{it} = s|\boldsymbol{y}) \approx \frac{1}{Q-M} \sum_{m=M+1}^{Q} I[S_{it}^{[m]} = s],$$

where the superscript $[m]$ denotes a draw from the posterior distribution of the variable, M is the size of the burn-in sample, Q is the total number of iterations of the Gibbs sampler, $I[\cdot]$ is an indicator function and $\boldsymbol{y}$ is the vector of all observed data. In Figure 5B we plot $P(S_{it} = s|\boldsymbol{y})$ versus t for each $s = 1, 2, 3$, and note $P(S_{it} = 3|\boldsymbol{y}) = 1$ if $y_{it} > 0$ as we know the disease is present. In Figure 6 (top) the maps show $P(S_{it} = 3|\boldsymbol{y})$ across i for various times, note $P(S_{it} = 3|\boldsymbol{y})$ when $y_{it} = 0$ gives the probability, given all observed data, that the disease is present and thus circulating undetected in area i at time t.

Finally, for the fitted values, we assumed a hypothetical new count y^*_{it} that is generated by the model assuming the same disease state, past counts and parameters that generated y_{it}. We can then draw from the posterior of the fitted value $y^{*[m]}_{it} \sim p(y^*_{it}|\boldsymbol{y})$ by drawing $y^{*[m]}_{it}$ from a $NB(\lambda^{[m]}_{it}, r^{[m]})$ if $S^{[m]}_{it} = 3$ and setting $y^{*[m]}_{it} = 0$ otherwise, for $m = M+1, \ldots, Q$. Figure 5A shows the posterior mean and 95% credible interval of y^*_{it}, approximated by such draws, against the observed value y_{it}, versus t. We compared the simulated values from the model with the observed values to assess the fit of the model, which is common in Bayesian statistics and is often referred to as a "posterior predictive check" (see (4) Section 5.6 for instance).

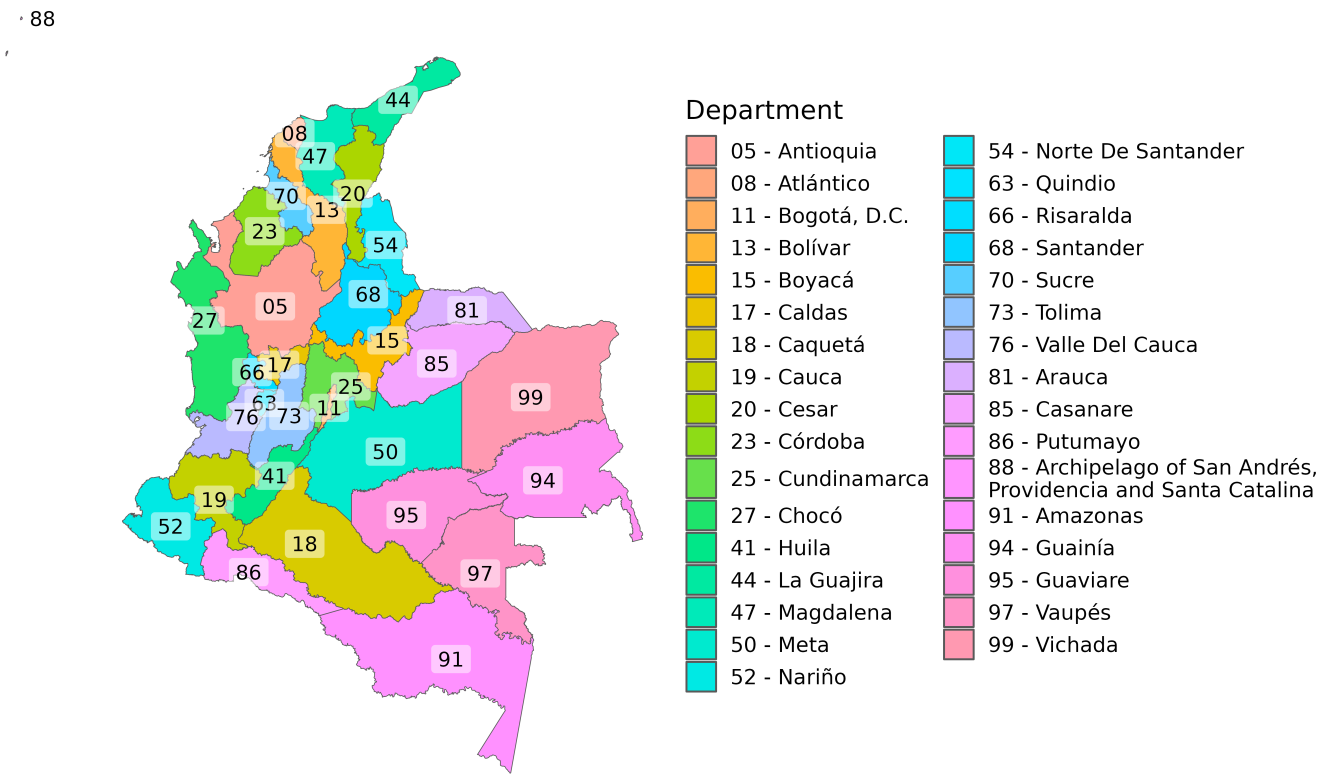

Figure S1. Departments of Colombia. Data source: Colombian National Administrative Department of Statistics - *Departamento Administrativo Nacional de Estadística* (DANE). Map created using R (version 4.3.2, https://www.r-project.org/).

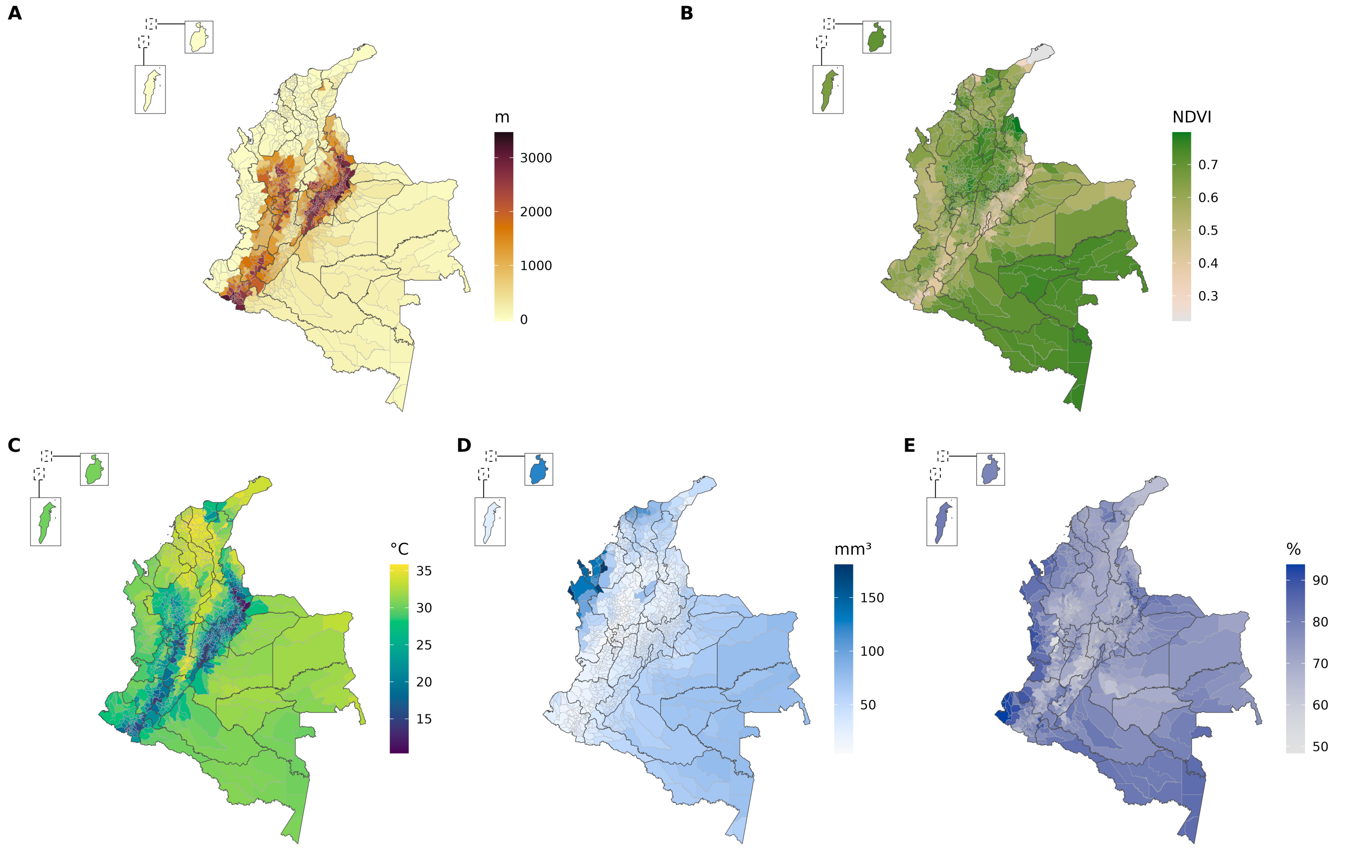

Figure S2. Distribution of covariates by municipality, epidemiological weeks 22/2015 to 39/2016, Colombia. (A) Elevation, (B) mean Normalized Difference Vegetation Index (NDVI), (C) mean maximum temperature, (D) total accumulated precipitation, and (E) mean relative humidity. Data sources: Colombian National Administrative Department of Statistics - *Departamento Administrativo Nacional de Estadística* (DANE); Siraj et al. (2019) (9). Maps created using R (version 4.3.2, https://www.r-project.org/).

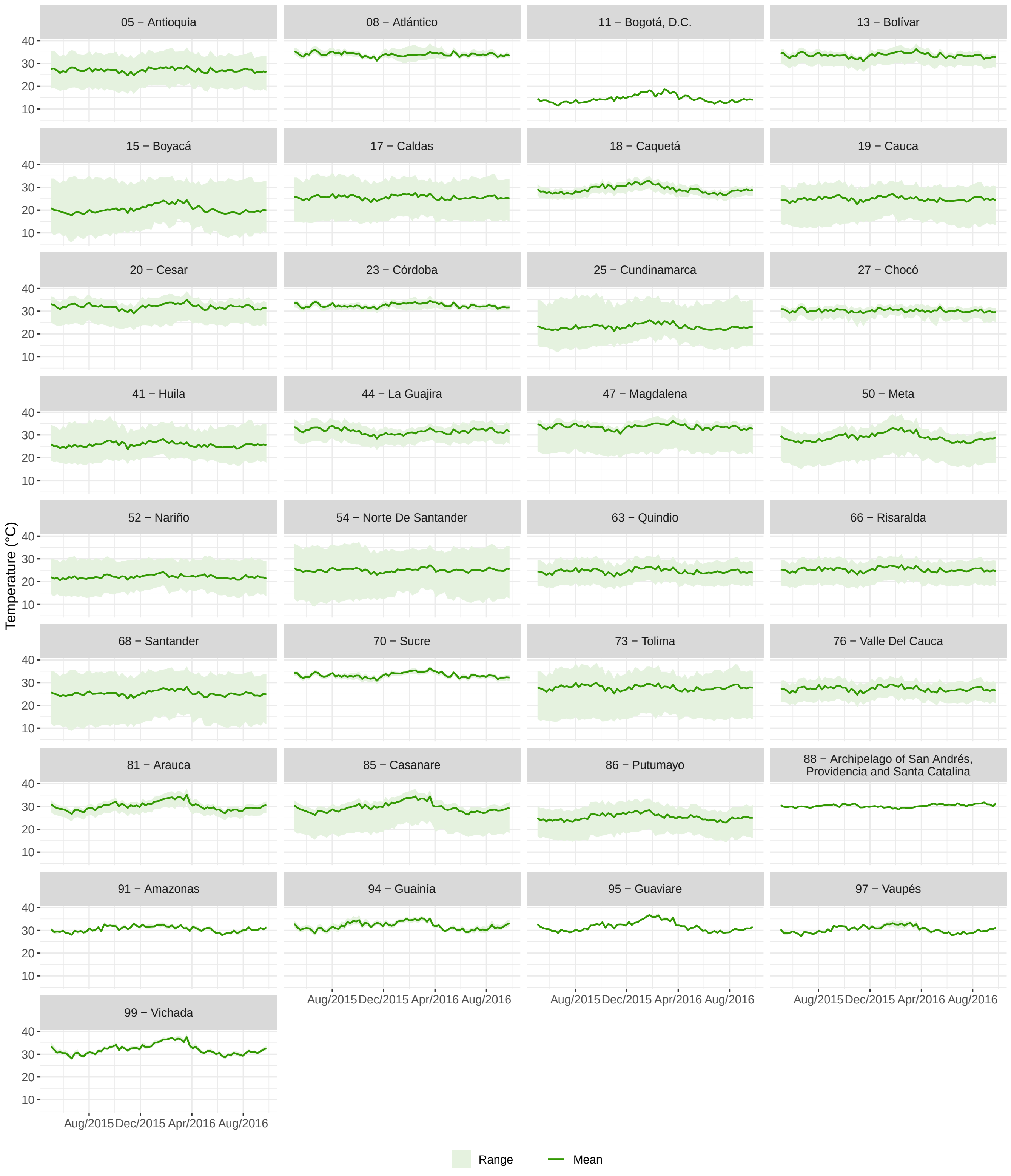

Figure S3. Maximum temperature (in °C) by department and epidemiological week (EW), EWs 18/2015 to 39/2016, Colombia. From the data by municipality, we calculated the mean and the range (minimum and maximum values) for each department. Data source: Siraj et al. (2019) (9).

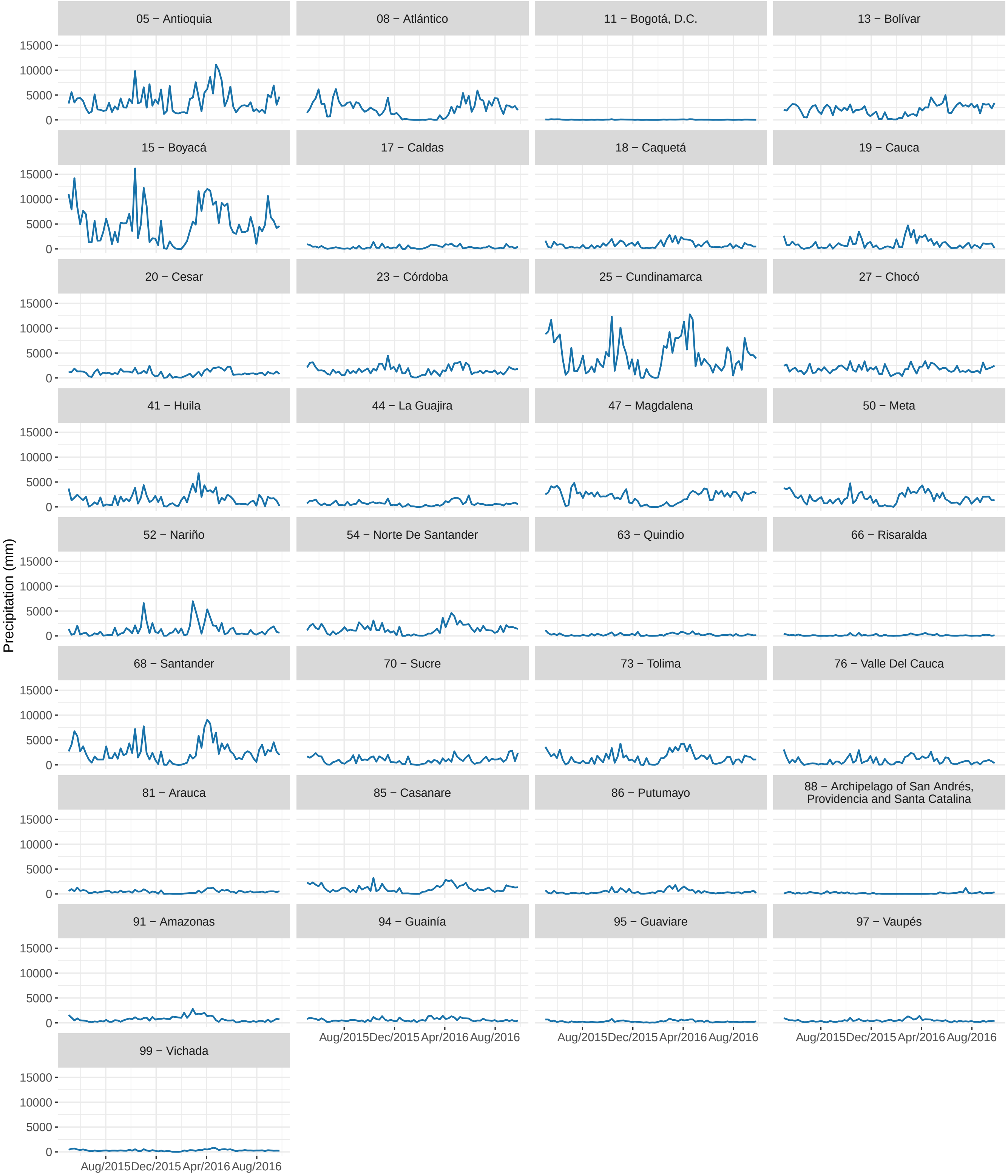

Figure S4. Accumulated weekly precipitation (in mm) by department and epidemiological week (EW), EWs 18/2015 to 39/2016, Colombia. The precipitation for each department was calculated by summing the data by municipality. Data source: Siraj et al. (2019) (9).

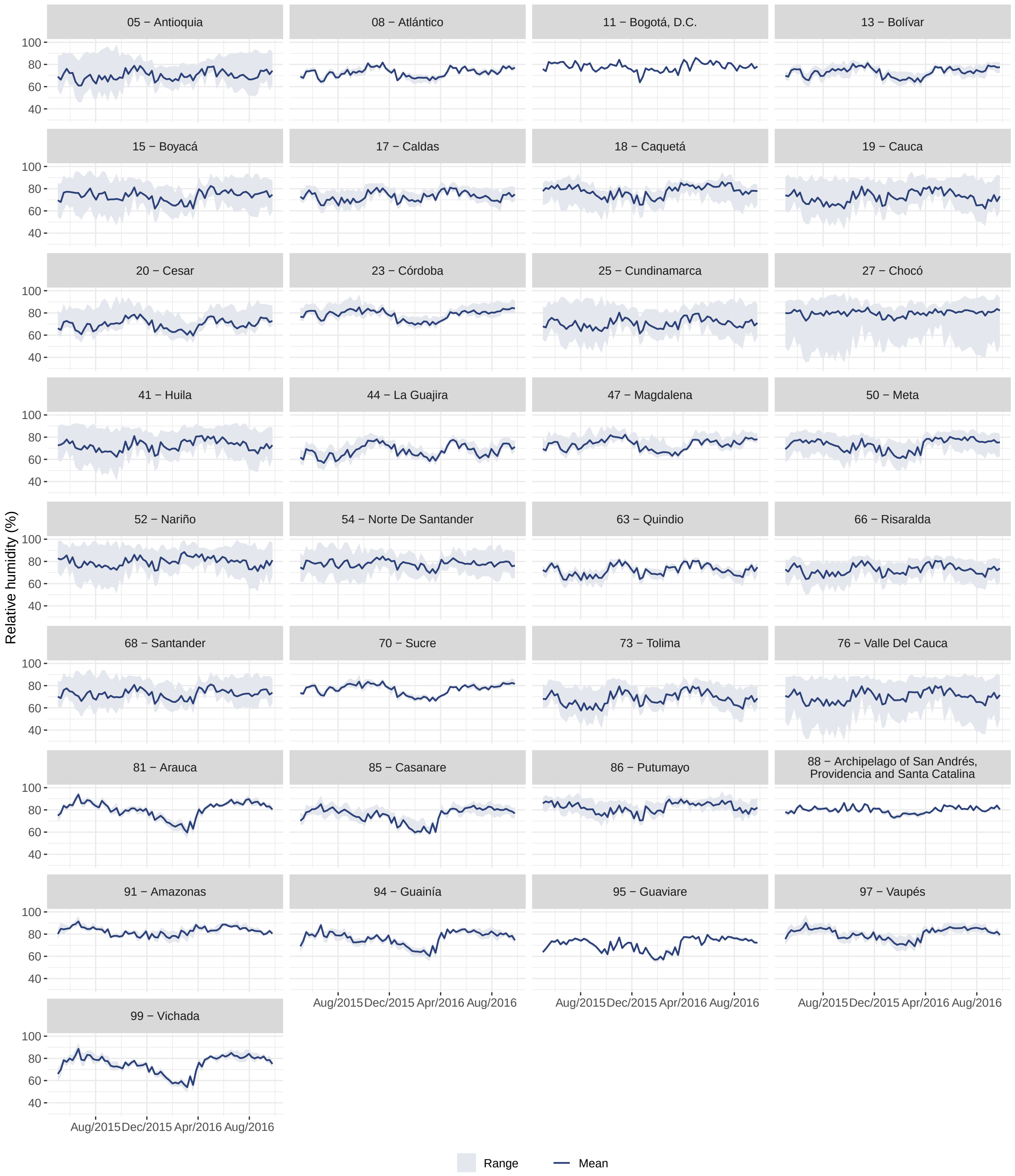

Figure S5. Relative humidity (%) by department and epidemiological week (EW), EWs 18/2015 to 39/2016, Colombia. From the data by municipality, we calculated the mean and the range (minimum and maximum values) for each department. Data source: Siraj et al. (2019) (9).

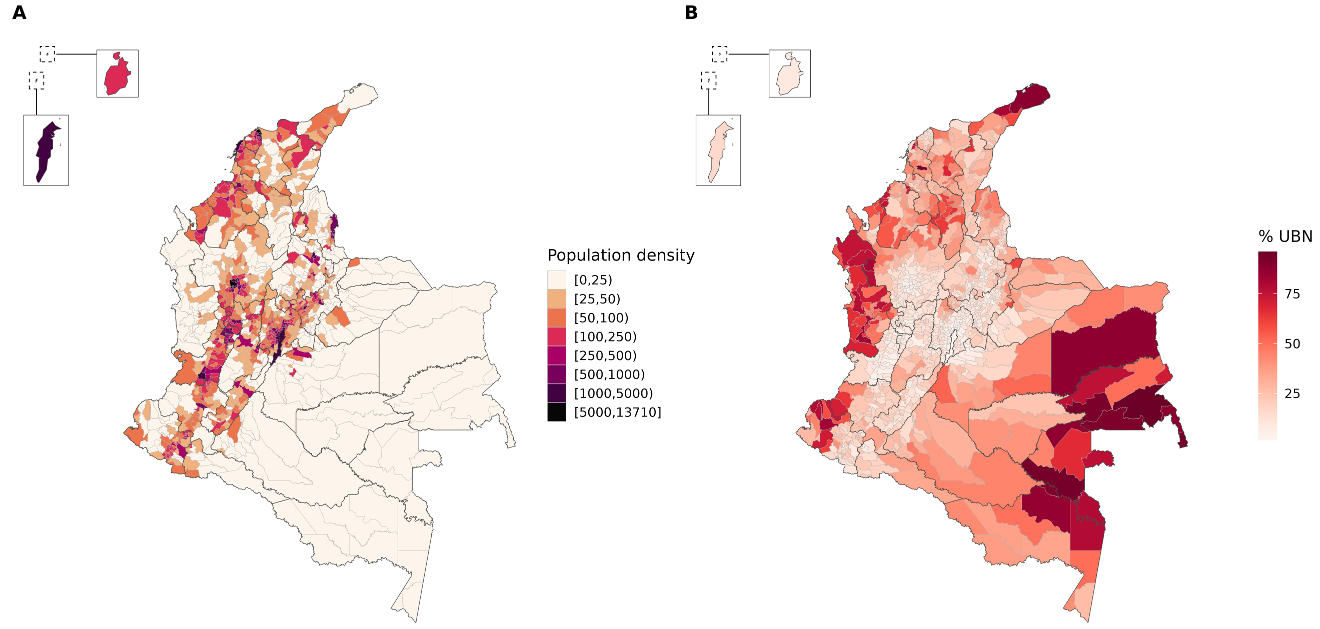

Figure S6. (A) Mean population density (2015-2016) by km^2 and (B) the percentage of population with unsatisfied basic needs (UBN) (2018) by municipality, Colombia. Data source: Colombian National Administrative Department of Statistics - *Departamento Administrativo Nacional de Estadística* (DANE). Maps created using R (version 4.3.2, https://www.r-project.org/).

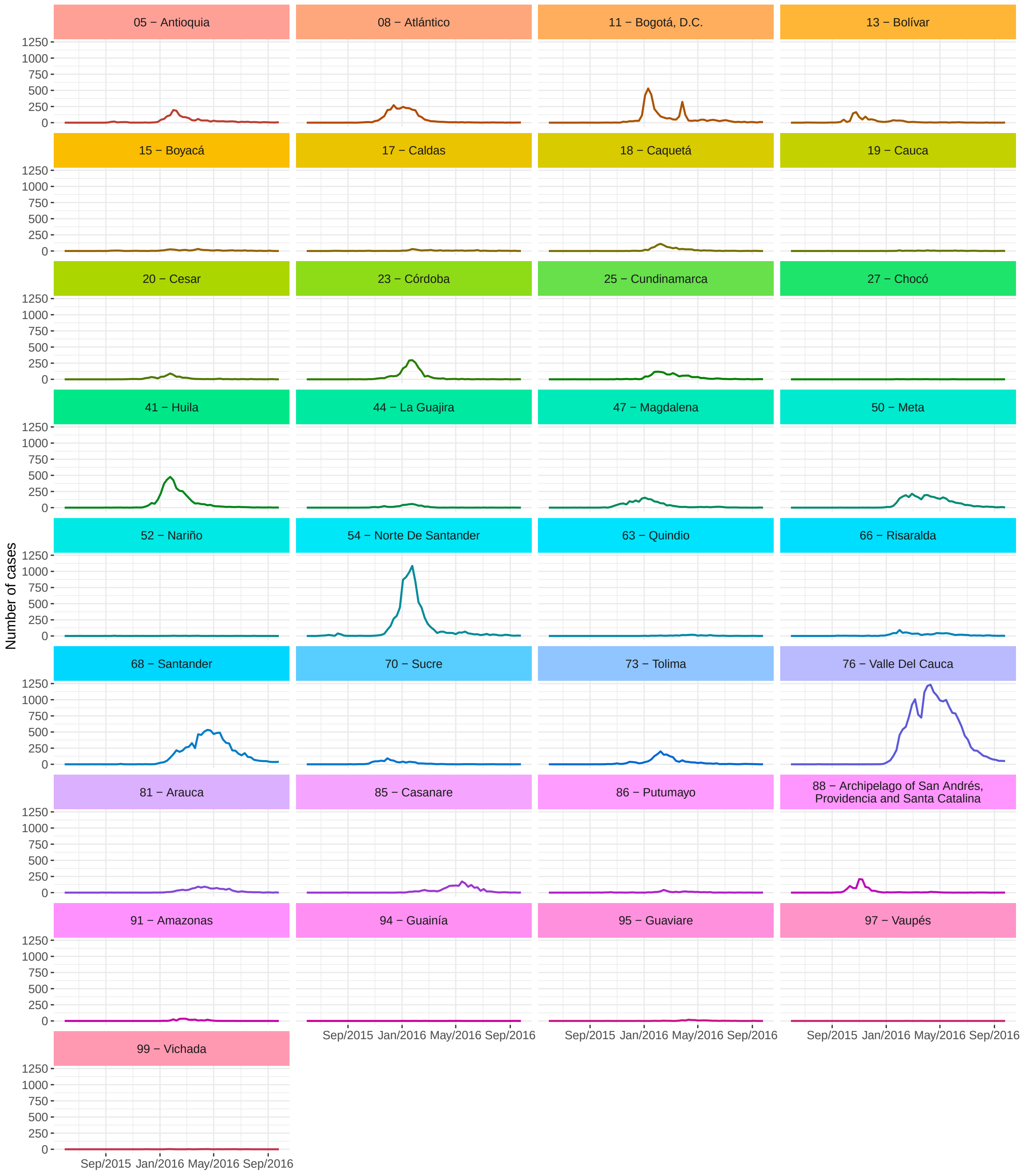

Figure S7. Number of reported Zika cases by department of residence and epidemiological week (EW) of first symptoms, EWs 22/2015 to 39/2016, Colombia. Data source: Colombian National Public Health Surveillance System - *Sistema Nacional de Vigilancia en Salud Pública* (SIVIGILA).

Table S1. Number and percentage of municipalities by percentage of weeks with no reported Zika cases, EWs 22/2015 to 39/2016, Colombia.

Percentage of weeks with no reported cases	Municipalities N	%
[0]	16	1.43
(0-25)	2	0.18
[25-50)	29	2.59
[50-75)	107	9.54
[75-100)	619	55.22
[100]	348	31.04
	1121	100.00

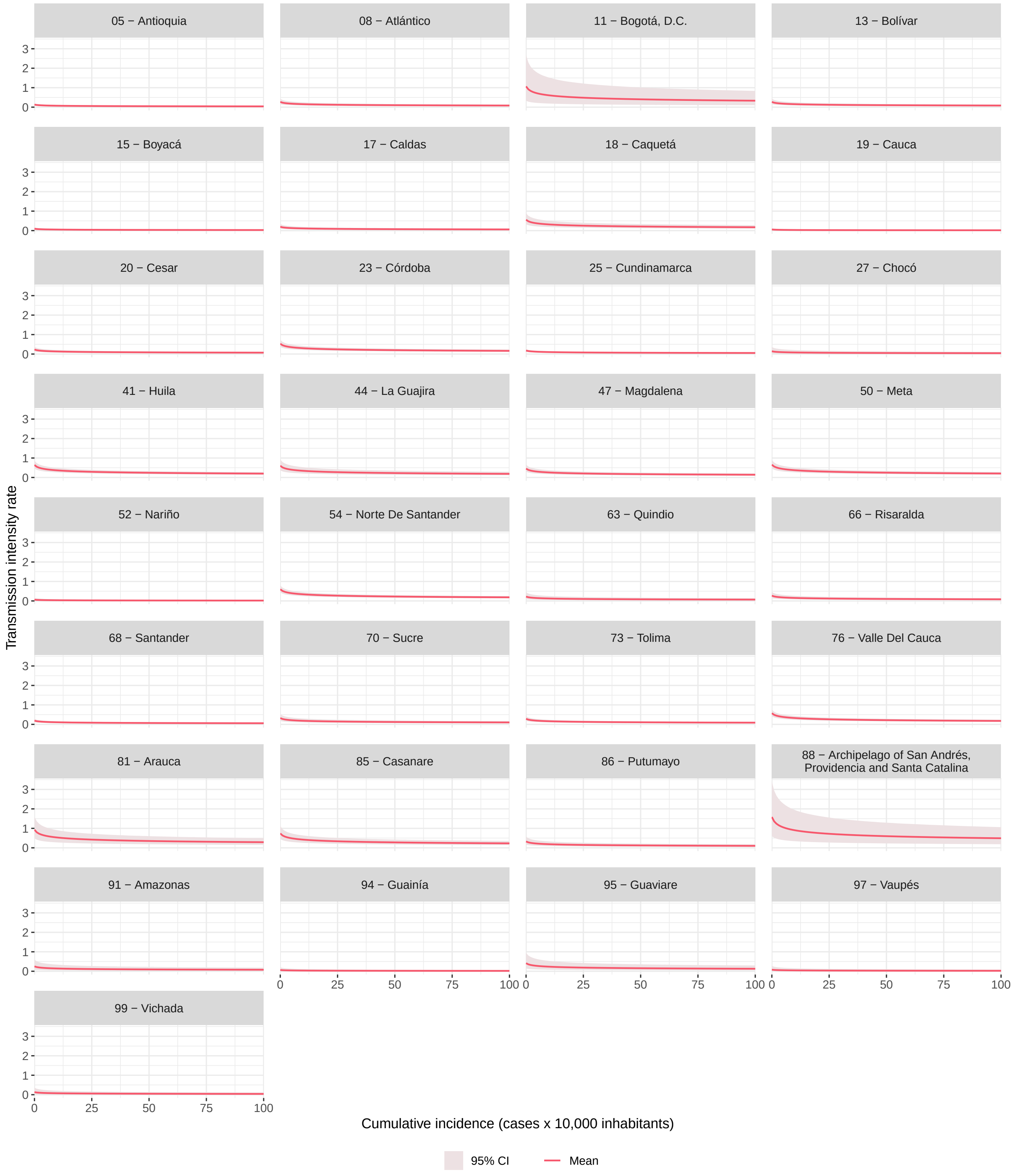

Figure S8. Cumulative incidence of reported Zika cases per 10,000 inhabitants (lagged by four weeks) association with the Zika transmission intensity rate by department of residence after adjusting for the department specific random effect and the average values of the other covariates in the department, epidemiological weeks 22/2015 to 39/2016, Colombia.

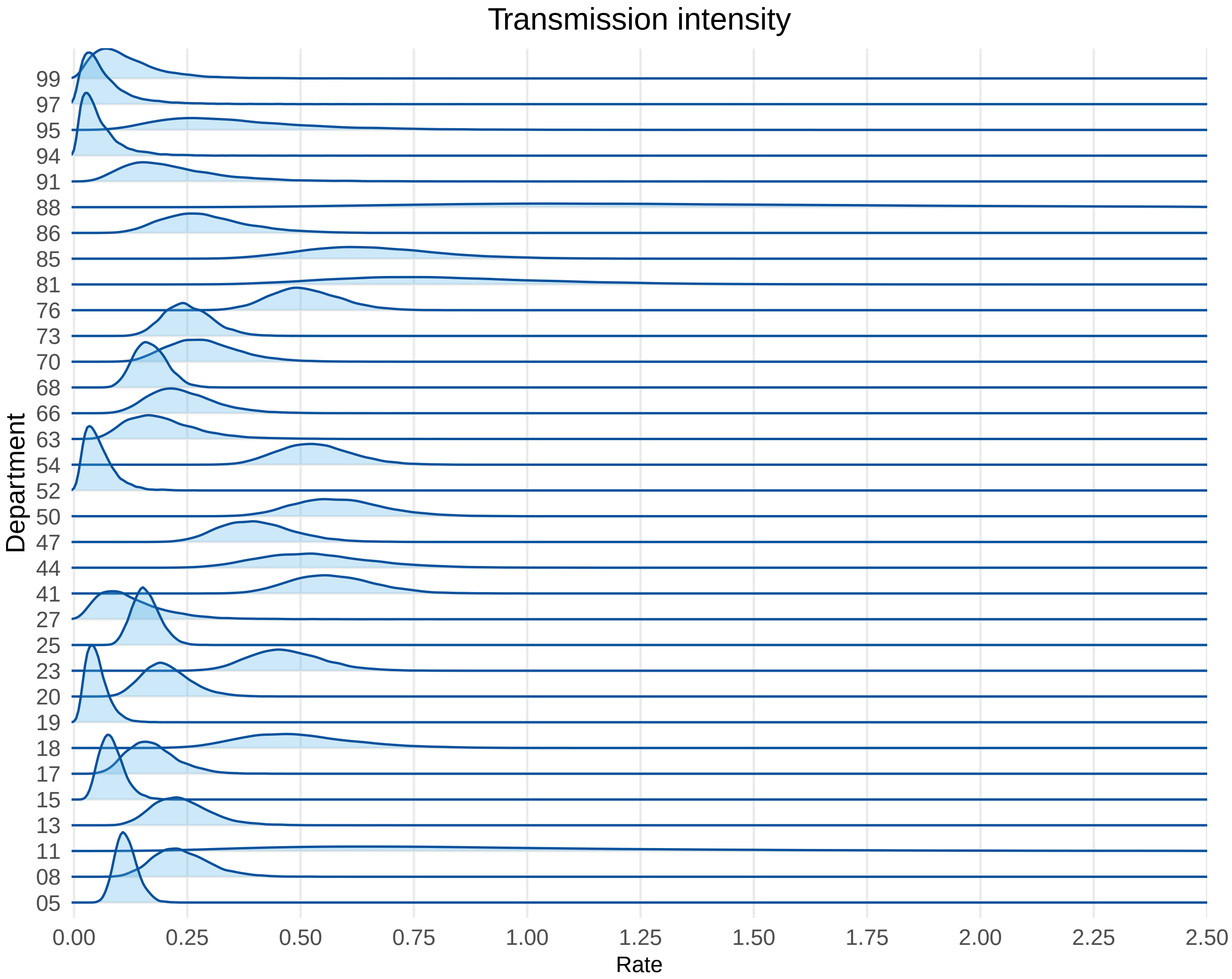

Figure S9. Estimated posterior distribution of the Zika transmission intensity rate by department after adjusting for the department specific random effect and the average values of the covariates in the department, epidemiological weeks 22/2015 and 39/2016, Colombia.

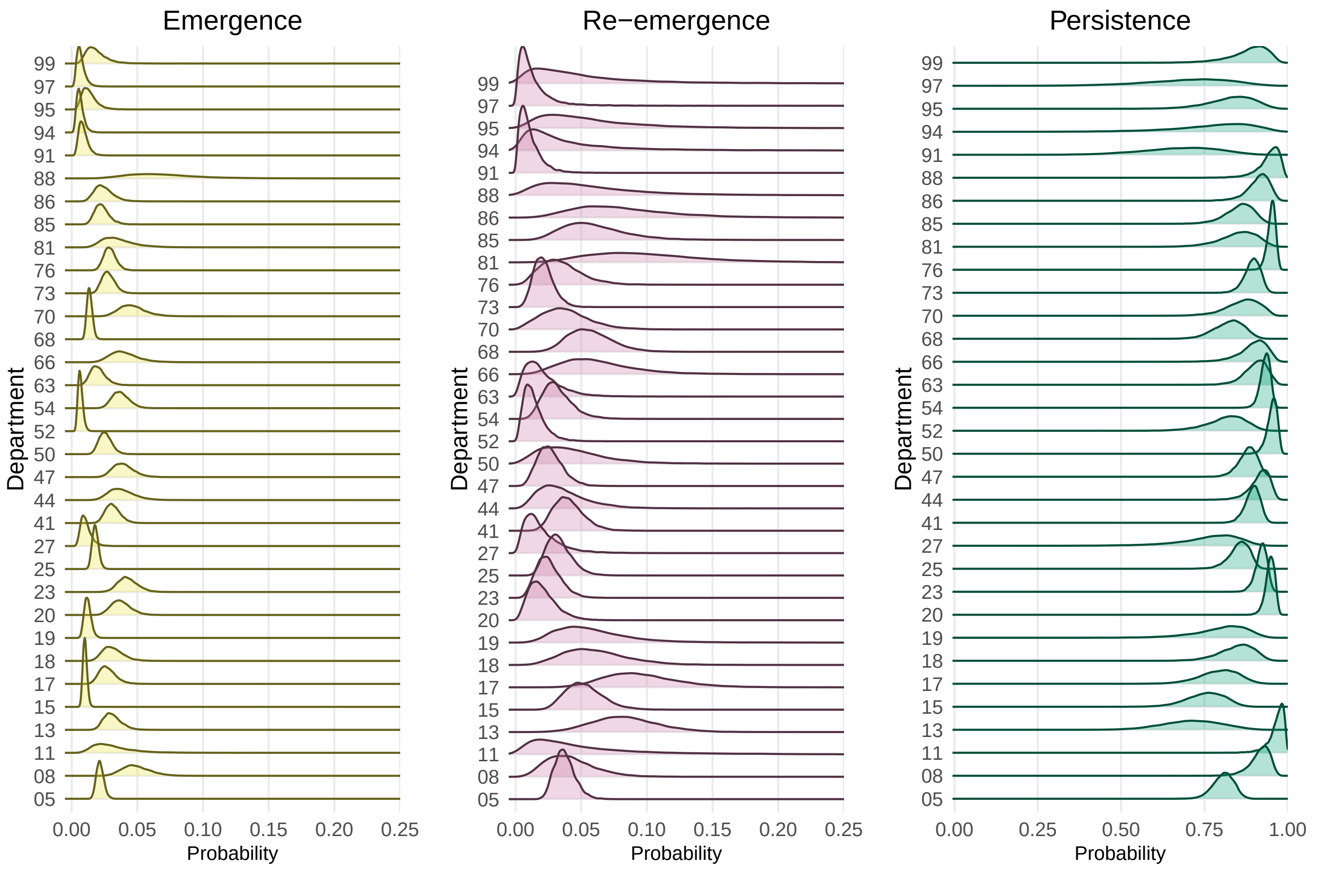

Figure S10. Estimated posterior distribution of the probability of emergence, re-emergence, and persistence of Zika by department after adjusting for the department specific random effect and the average values of the covariates in the department, epidemiological weeks 22/2015 and 39/2016, Colombia.

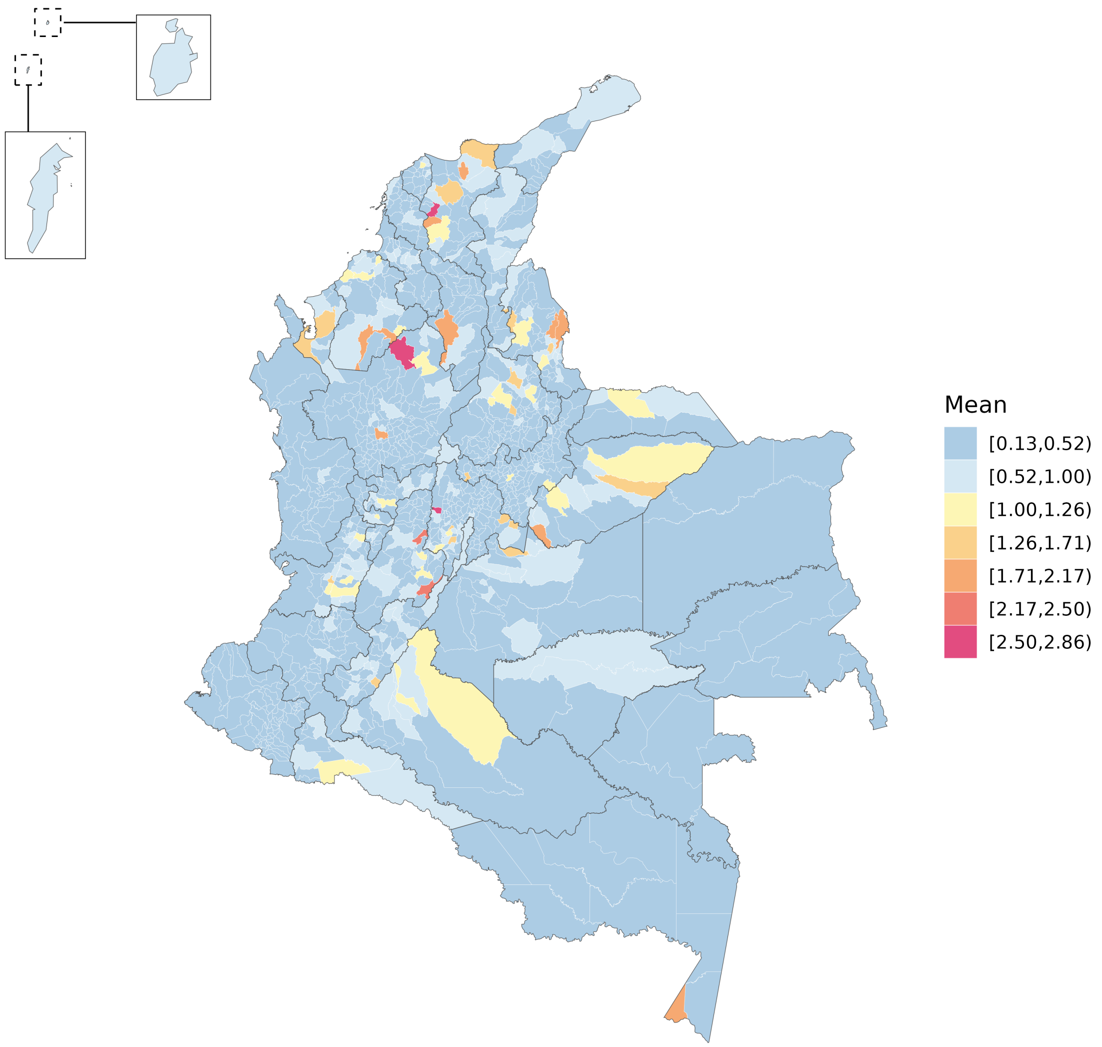

Figure S11. Estimated posterior mean baseline of the the expected reported cases by municipality, epidemiological weeks 22/2015 and 39/2016, Colombia. Map created using R (version 4.3.2, https://www.r-project.org/).

Movie S1. Space-time distribution of the estimated Zika transmission intensity rate by municipality and epidemiological week (EW), EWs 23/2015 to 39/2016, Colombia. Maps created using R (version 4.3.2, https://www.r-project.org/).

Movie S2. Space-time distribution of the estimated probability of Zika presence and of reported Zika cases by municipality and epidemiological week (EW), EWs 23/2015 to 39/2016, Colombia. Maps created using R (version 4.3.2, https://www.r-project.org/).

References

1. Pérez, N. T. Protocolo de vigilancia en salud pública – Enfermedad por Virus Zika (2017).
2. Douwes-Schultz, D. & Schmidt, A. M. Zero-state coupled Markov switching count models for spatio-temporal infectious disease spread. *J. Royal Stat. Soc. Ser. C (Applied Stat.* **71**, 589–612, DOI: 10.1111/rssc.12547 (2022).
3. de Valpine, P. *et al.* Programming with models: writing statistical algorithms for general model structures with NIMBLE. *J. Comput. Graph. Stat.* **26**, 403–413, DOI: 10.1080/10618600.2016.1172487 (2017).
4. Reich, B. J. & Ghosh, S. K. *Bayesian Statistical Methods* (CRC Press, 2019).
5. Plummer, M., Best, N., Cowles, K. & Vines, K. CODA: convergence diagnosis and output analysis for MCMC. *R News* **6**, 7–11 (2006). Number: 1.
6. Shaby, B. A. & Wells, M. T. Exploring an Adaptive Metropolis Algorithm (Department of Statistics, Duke University, 2010).
7. Chib, S. Calculating posterior distributions and modal estimates in Markov mixture models. *J. Econom.* **75**, 79–97, DOI: 10.1016/0304-4076(95)01770-4 (1996).
8. Frühwirth-Schnatter, S. *Finite Mixture and Markov Switching Models*. Springer Series in Statistics (Springer-Verlag, New York, 2006).
9. Siraj, A. S. *et al.* Data from: Spatiotemporal incidence of Zika and associated environmental drivers for the 2015-2016 epidemic in Colombia, DOI: 10.5061/DRYAD.83NJ1 (2019).
